# Medical Obituaries

**Published:** 1853-11

**Authors:** 


### MEDICAL OBITUARIES.

**M. Orfilla**, the distinguished chemist and toxicologist, died at Paris, on the 12th of March, of Pneumonia. He had for a long series of years held a most prominent position, before the world, as an analytical chemist, and his name is associated, in every climate of the globe, with the most brilliant researches respecting poisons. He had recently been much afflicted, in consequence of the epilepsy of a son, who was confined in a lunatic asylum. He was so well on the 4th of March that he lectured to a large audience, in his usual health; but was attacked with Pneumonia and died on the 12th.

---

ROBERT J. GRAVES, M. D., died at Dublin, Ireland, on the 20th March, aged 57 yrs. Dr. G. had of late years been a victim to the gout, but his last illness involved the lungs. He had become celebrated throughout the world, as a physician of eminent ability; he held a high rank as a medical author, and his works are characterized by an originality and force of thought rarely exhibited in writings of that class. His loss is deeply regretted by the profession in every country.

---

WM. BEAUMONT, M. D., died at his residence in St. Louis, April 25, in the 68th year of his age. He was a native of Connecticut; studied medicine at St. Albans, Vermont, and afterwards joined the Army, as Asst. Surgeon, and continued with it for more thant wenty years. He was most widely celebrated for his admirable and original treatise, "The Physiology of Digestion, and Experiments on the Gastric Juice," based upon experiments conducted by himself upon a Canadian, Alexis St. Martin, whom he attended upon the northern frontier, in 1825. Dr. B. had resided for many years at St. Louis, where he was most widely and favorably known, as a learned and skilful physician, and a kind hearted, generous man.

---

PROF. N. CHAPMAN, M. D., died in Philadelphia, after a long sickness, July 1st; aged 73 yrs. Dr. C. received his early education at the Academy of Alexandria, founded by Gen. Washington, where he passed six years. He came to Philadelphia in 1797, and began the study of medicine with the late Prof. Rush, of whom he was a favorite pupil. In 1801 he went abroad, and spent four years, chiefly in Edinburgh and London, where he was the private pupil of Abernethy. After his return, he settled in Philadelphia, became immediately very popular,—was appointed adjunct Prof. of Midwifery in the University of Pennsylvania, and at the death of Rush, in 1812, was transferred to the Chair of Theory and Practice, which he filled till he resigned in 1850. He was one of the earliest and most successful of American Medical Authors; held a very eminent position in his profession; was widely known throughout the union, and was chosen first President of the American Medical Association.

PROF. CHARLEL CALDWELL, M. D., died at Louisville, Ky., on the 9th of July; aged nearly 90 years. He was born in N. Carolina, of humble parentage, and relied on himself for his advancement. He was a student of Dr. Rush, and graduated at the University of Pennsylvania, in 1796. While a student he translated, with notes, from the Latin, Blumenbach's Elements of Physiology, in two octavo vols., with an original appendix on "Animal Electricity," which ably predicted his future eminence as a medical author. In 1801 he published a work upon "the Epidemics of the United States." In 1819 he was appointed Prof. of Institutes of Medicine in the Medical Department of Transylvania University, where he labored till 1837, when he accepted the same Chair in the "Louisville Medical Institute," now the Medical Department of the University of Louisville. He was a prominent physician, a man of energy and industry, of great learning and eloquence.

